# Homogeneous Nature of Malaysian Marine Fish *Epinephelus fuscoguttatus* (*Perciformes; Serranidae*): Evidence Based on Molecular Markers, Morphology and Fourier Transform Infrared Analysis

**DOI:** 10.3390/ijms160714884

**Published:** 2015-07-02

**Authors:** A’wani Aziz Nurdalila, Hamidun Bunawan, Subbiah Vijay Kumar, Kenneth Francis Rodrigues, Syarul Nataqain Baharum

**Affiliations:** 1 Institute of Systems Biology, Universiti Kebangsaan Malaysia, UKM Bangi, 43600 Selangor, Malaysia; E-Mails: nurdalila26@gmail.com (A.A.N.); hamidunb@yahoo.com (H.B.); 2 Biotechnology Research Institute, Universiti Malaysia Sabah, Jalan UMS, 88400 Kota Kinabalu Sabah, Malaysia; E-Mails: vijay@ums.edu.my (S.V.K.); kennethr@ums.edu.my (K.F.R.)

**Keywords:** 16S, cytochrome oxidase, species identification, *Epinephelus fuscoguttatus*, *Epinephelus hexagonatus*

## Abstract

Taxonomic confusion exists within the genus *Epinephelus* due to the lack of morphological specializations and the overwhelming number of species reported in several studies. The homogenous nature of the morphology has created confusion in the Malaysian Marine fish species *Epinephelus fuscoguttatus* and *Epinephelus hexagonatus*. In this study, the partial DNA sequence of the 16S gene and mitochondrial nucleotide sequences of two gene regions, Cytochrome Oxidase Subunit I and III were used to investigate the phylogenetic relationship between them. In the phylogenetic trees, *E. fuscoguttatus* was monophyletic with *E. hexagonatus* species and morphology examination shows that no significant differences were found in the morphometric features between these two taxa. This suggests that *E. fuscoguttatus* is not distinguishable from *E. hexagonatus* species, and that *E. fuscoguttatus* have been identified to be *E. hexagonatus* species is likely attributed to differences in environment and ability to camouflage themselves under certain conditions. Interestingly, this finding was also supported by Principal Component Analysis on Attenuated Total Reflectance–Fourier-transform Infrared (ATR-FTIR) data analysis. Molecular, morphological and meristic characteristics were combined with ATR-FTIR analysis used in this study offer new perspectives in fish species identification. To our knowledge, this is the first report of an extensive genetic population study of *E. fuscoguttatus* in Malaysia and this understanding will play an important role in informing genetic stock-specific strategies for the management and conservation of this highly valued fish.

## 1. Introduction

Groupers (*Epinephelinae* spp., Serranidae) are an important marine fish in the marine culture industry since they are easily bred in captivity. Groupers are highly prized and sought after since they have a higher market value compared to other marine fishes [1]. The fish are wildly distributed and can be found in the in the Atlantic, Mediterranean and Indo-Pacific region, including the Red Sea [2].

Grouper farming appears to have great promise for commercialization, and coastal cage culture has the potential for continued development. However, the unreliable and limited supply of grouper fry has hindered the growth of the grouper industry. For sustainable growth, the production of the fish fry in a controlled hatchery setting is needed to lessen the demand for supply from the wild [3]. Furthermore, precise taxonomic and species identification is essential for the appropriate managing of fishery resources.

Incorrect identifications of waste resources have cost the agricultural sector many millions of pounds, and have invalidated the results of entire research programmes. Kuo *et al.*, [4] also reported that inbreeding was expected to cause defective recessive alleles that will reduce trait qualities and also survival rates. These can result in growth depression due to the sensitivity to environmental stress, which can lead to trait depression, compromising the animals’ fitness and disease resistance. As a result, the farmers may observe poor survival and growth of the seed and wild brood stock since some fishes are a low value species that were formerly not considered as food fish [5].

Under field conditions and natural habitat, the identification and diagnostic methods to distinguish the grouper species are typically based on the colour pattern and morphological characters. In the genus *Epinephelus*, taxonomic confusion often occurred to identify individual species due to lack of morphological distinctive characters and specializations as well as the overwhelming number of the species [6]. The identification of the grouper species in this genus is normally based on colour configuration and geographic zone, even if it is not really effective to discriminate among grouper genera by geographic locality [6].

Genetic markers such as the Cytochrome b (Cyt b) sequence and 16s rRNA have proven to be informative to facilitate the investigation of deeper evolutionary relationships in the genetic structure and gene flow and can improve species identification within members of the genus *Epinephelus* [6,7,8]. The first comprehensive molecular and phylogenetic relationship study on the genus *Epinephelus* was reported by Craig *et al.* [6] using 16S rDNA. The study suggested that the genus *Epinephelus* is paraphyletic by forming two distant clades. Further study on molecular phylogeny of the subfamily Epinephelinae was reported by Craig and Hasting [9] using two nuclear and two mitochondrial genes and confirmed the monophyly of the genera *Cephalopholis*, *Epinephelus* and *Mycteroperca*.

Previously, we reported a study identifying *E. fuscoguttatus* and *E. hexagonatus* using the Cyt b gene to construct phylogenetic tree. Based on the previous results, the phylogenetic tree of Maximum Parsimony (MP), Molecular Evolution (ME) and Neighbor-Joining (NJ) showed that the group cluster of the trees have a mix of individuals of those two different species [10]. In this study, we used the 16s rRNA, Cytochrome Oxidase Subunit I (COI) and III (COIII) genes as molecular markers to study the Malaysian marine fishes *Epinephelus fuscoguttatus* and *Epinephelus hexagonatus*. *Epinephelus fuscoguttatus* (Forsskal, 1775), also known as Brown-marbled grouper, locally known as the Tiger grouper and *Epinephelus hexagonatus* (Forster, 1801), known as Starspotted grouper. *Epinephelus* hexagonatus have been observed in several locations in Peninsular Malaysia and Borneo. Both of them have similar characteristics such as a black spot on the upper caudal peduncle, dark brown circular spots, and head, jaws, and gills cover with spots, however we believe that this is probably due to environment or the homogeneous nature of the morphology. In order to identify these two taxa, we performed molecular identification, morphology characterization and Attenuated Total Reflectance-Fourier-transform Infrared (ATR-FTIR) data analysis to solve this mystery.

## 2. Results

The 16S and Cytochrome Oxidase Subunit I and III genes were partially sequenced from 125 samples. The number of base substitutions per site is based on the pairwise analysis of 16S, COI and COIII haplotypes. The evolutionary history was determined using the ME (data not shown), and MP (data not shown) also NJ methods (data are shown). The ME, MP, and NJ trees showed similar pattern for the 16S, COI and COIII genes.

### 2.1. Phylogenetic Trees

**Figure 1:**
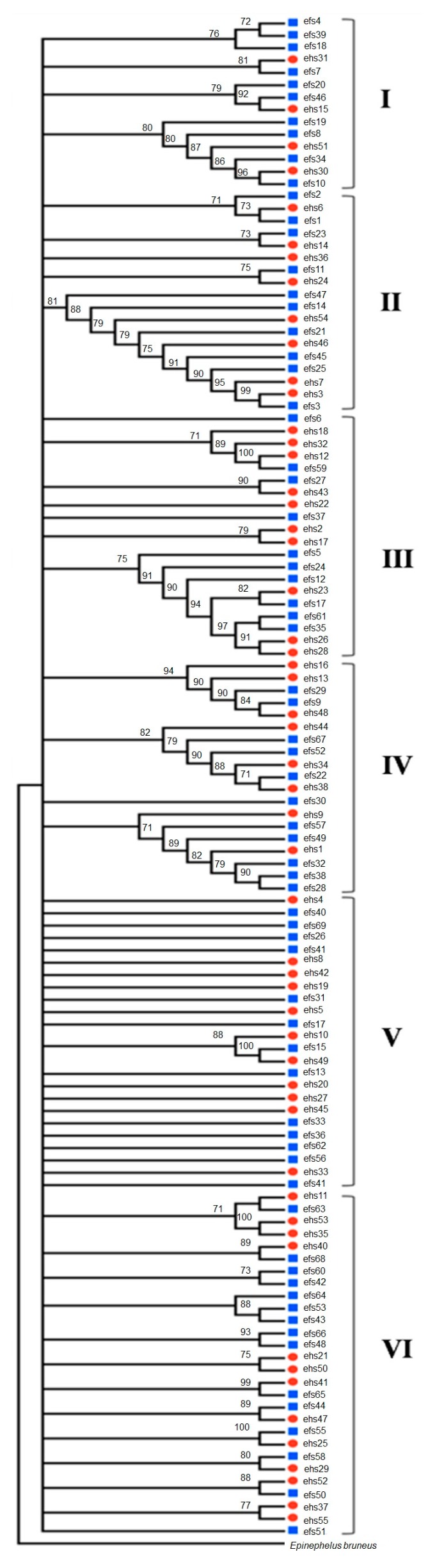
NJ evolutionary of 16S gene. The square shapes represent *E. fuscoguttatus*, and the round shapes represent *E. hexagonatus*. Numbers represent grouper cluster. Branches corresponding to partitions reproduced in less than 70% of the bootstrap replicates are collapsed. I–VI represent six unique of haplotypes.

For the 16S gene, the bootstrap consensus NJ tree evolutionary relationships (Figure 1) determined from 1000 replicates to represent the evolutionary history of the taxa analysed. From the total of 125 samples that were successfully sequenced, six unique haplotypes were found in the analysis. The consistency index is 0.782609; the retention index is 0.871795; and the composite indices are 0.815911 and 0.782274 for all sites and parsimony-informative sites, respectively. Branches corresponding to partitions reproduced in less than 70% of the bootstrap replicates are collapsed [11]. The tree is drawn to scale. Branch lengths were calculated using the average pathway method and are in units of the number of changes over the entire sequence. The statistical values in genetic distances among the grouper samples ranged from 0.009 to 0.015. Out of 125 samples, 54 were identified as *E. hexagonatus* and 71 were identified as *E. fuscoguttatus*.

The sequences of COI were deposited in GenBank with accession numbers JN674159 to JN674166. The number of base substitutions per site is based on the pairwise analysis of 76 sequences. The evolutionary history was inferred using the MP, ME, and NJ methods. The ME optimal tree branch length is 0.94837547. The percentage of replicate trees in which the associated taxa clustered together in the bootstrap test (1000 replicates) is shown next to the branches. The phylogenetic tree was linearised assuming equal evolutionary rates in all lineages. We did not observe major changes in the genealogy of the *E. fuscoguttatus* and *E. hexagonatus* sequences.

For the MP method (data not shown), the consensus tree inferred from the 65 most parsimonious trees is shown. Branches corresponding to partitions reproduced in less than 70% of trees are collapsed. For parsimony-informative sites, the consistency index is 0.917730, the retention index is 0.803582, and the composite index is 0.716039. For all sites, the composite index is 0.748753. The percentage of parsimonious trees in which the associated taxa clustered together is shown next to the branches. The evolutionary history was inferred using the NJ method. The NJ optimal tree branch length is 0.91644275 (data not shown). The percentage of replicate trees in which the associated taxa clustered together in the bootstrap test (1000 replicates) is shown next to the branches. The phylogenetic tree was linearised assuming equal evolutionary rates in all lineages.

An average of 1500 nucleotide base pairs per taxon was amplified from DNA of *E. fuscoguttatus* and *E. hexagonatus* by employing the COIII primer pairs. No insertions/deletions or stop codons were observed with high sequence similarity (98%–100%) to their respective species data in GenBank. Thus, the correct identity of these species was confirmed. The sequences of COIII were deposited in GenBank with accession numbers JN859014 to JN859041. The evolutionary history of the COIII gene was also inferred using the MP, ME, and NJ methods. The ME optimal tree branch length is 0.75335376. The percentage of replicate trees in which the associated taxa clustered together in the bootstrap test (1000 replicates) is shown next to the branches. The tree is drawn to scale, with branch lengths in the same units as those of the evolutionary distances used to infer the phylogenetic tree. The transition/transversion ratio, base frequencies, and gamma distribution shape parameter as estimated were Ti/Tv = 8.7879; A = 0.2885, C = 0.3589, G = 0.1481, and T = 0.2044; and α = 2.5876, respectively. For the MP tree, the consistency index is 0.725490, the retention index is 0.810902, and the composite index is 0.835294 for the parsimony-informative sites. The composite index is 0.764407 for all sites (data not shown).

The optimal neighbour-joining tree a branch length is 0.75584609 (data not shown). The percentage of replicate trees in which the associated taxa clustered together in the bootstrap test (1000 replicates) is shown next to the branches. These results indicate that the topological structures of the two trees were nearly identical. They showed relatively close relationships among the species groups and were strongly supported by bootstrap values.

### 2.2. Morphological Analysis

**Figure 2:**
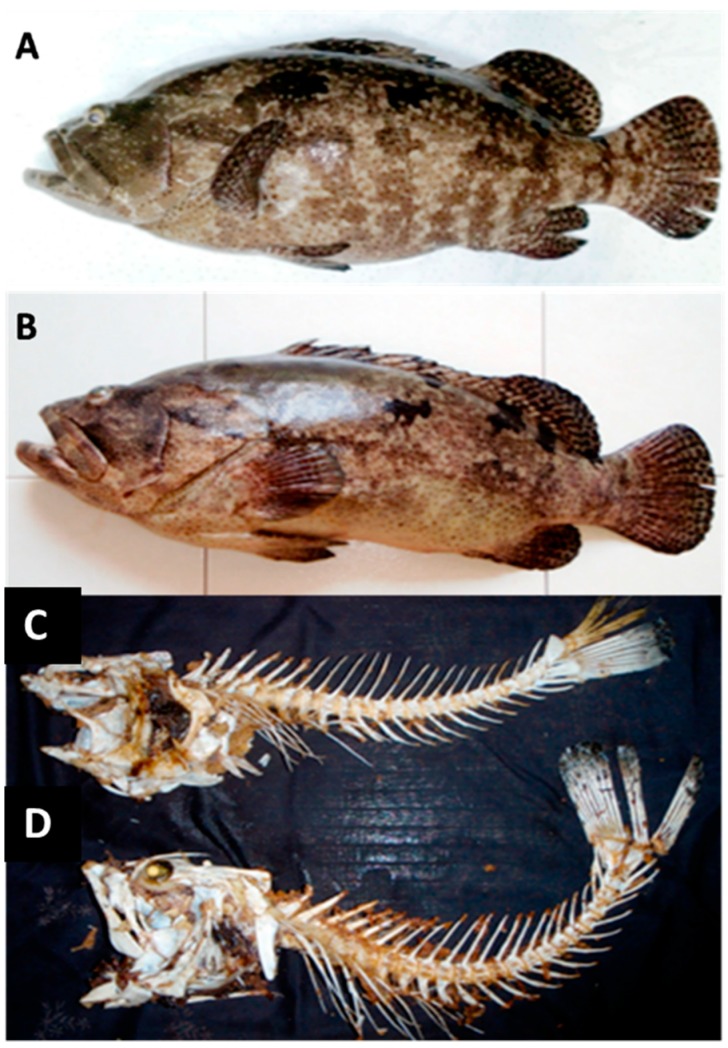
Morphology, morphometric and meristic characteristics (**A**) *Epinephelus hexagonatus*; (**B**) *Epinephelus fuscoguttatus*; (**C**) Bones of *Epinephelus hexagonatus* and (**D**) Bones of *Epinephelus fuscoguttatus*.

Ten specimens of *E. fuscoguttatus* and *E. hexagonatus* (Figure 2) from Terengganu and Borneo, ranging from 58.0 to 61.0 cm total length (TL) and 6.0 to 8.5 kg body weight (BW), were used for morphometric and meristic characteristic analyses (Figure 2). The main morphometric and meristic data are reported in Table 1 and Table 2, respectively. Fork length (FL), standard length (SL), head length (HL), caudal fin length (CFL), dorsal fin length (DFL), pectoral fin length (PFL), anal fin length (AFL), mouth length (ML), snout length (SnL), body width length (BwL), pelvic fin ray, first dorsal fin ray, second dorsal fin ray, anal fin ray, caudal fin ray and pectoral fin ray were examined. Body depth was 2.6 to 2.9 times in standard length and the lateral- body scales of fish is approximately more than 10 cm standard length smooth, with auxiliary scales of shape.

**Table 1:** Definitions of morphometric measurements and meristic counts used in this study.

Characters	Description
***11 Morphometrics***	
Total length (TL)	Tip of the lower jaw to the end of caudal fin
Fork length (FL)	Tip of the upper jaw to the tail base
Standard length (SL)	Tip of the upper jaw to the end of caudal fin
Head length (HL)	From the front of the upper lip to the posterior end of the opercular
Caudal fin length (CFL)	From tail base to tip of the caudal fin
Dorsal fin length (DFL)	Front of the upper lip to the origin of the dorsal fin
Pectoral fin length (PFL)	From base to tip of the pectoral fin
Anal fin length (AFL)	Front of the upper lip to the origin of the anal fin
Mouth length (ML)	Straight line measurement between the snout tip and posterior
Snout length (SnL)	The front of the upper lip to the flesh anterior edge of the orbit
Body width length (BwL)	The greatest width just posterior to the gill opening
***6 Meristic***	
Pelvic fin ray	Number of soft fin rays in the pelvic fin
1st dorsal fin ray	Number of soft fin rays in 1st dorsal fin
2nd dorsal fin ray	Number of soft fin rays in 2nd dorsal fin
Anal fin ray	Number of soft fin rays in anal fin Caudal fin ray
Anal fin ray	Number of soft fin rays in anal fin Caudal fin ray
Pectoral fin ray	Number of soft fin rays in pectoral fin

**Table 2:** Morphometric and meristic measurements.

Morphometrics					Meristics								
Measurements (cm)	Min		Max/Mean		Mean ± SD		TL (%)		Measurement	Range		Mean ± SD	
	EF	EH	EF	EH	EF	EH	EF	EH		EF	EH	EF	EH
Total length (TL)	58	58	61	59	59.5 ± 1.13	58.5 ± 1.21							
Fork length (FL)	50.5	49.4	53.7	53.3	52.3 ± 1.31	51.4 ± 0.89	87.8	87.8	Pelvic fin ray	6 to 7	6 to 7	6.5 ± 0.53	6.5 ± 0.5
Standard length (SL)	48.3	45.2	51.7	52	50.1 ± 1.18	48.6 ± 1.03	84	83.1	1st dorsal fin ray	11 to 12	11 to 12	11.6 ± 0.52	11.5 ± 0.5
Head length (HL)	14	14	15.5	14.8	14.8 ± 0.47	14.4 ± 0.58	24.9	24.6	2nd dorsal fin ray	9 to 13	9 to 14	11.1 ± 1.73	11.5 ± 1.78
Caudal fin length (CFL)	5.7	5.5	6.4	7.1	6.07 ± 0.25	6.3 ± 0.31	10.2	10.8	Anal fin ray	7 to 9	7 to 8	8 ± 0.82	7.5 ± 0.73
Dorsal fin length (DFL)	13.2	14.2	16.4	15.8	15.0 ± 1.13	15 ± 1.03	25.3	25.6	Caudal fin ray	14 to 15	14 to 15	14.5 ± 0.53	14.5 ± 0.42
Pectoral fin length (PFL)	8.2	8.2	9.5	9.5	8.85 ± 0.43	8.85 ± 0.4	14.9	15.1	Pectoral fin ray	18 to 20	17 to 20	18.8 ± 0.92	18.5 ± 1.13
Anal fin length (AFL)	7.9	7.5	11.3	10.8	9.62 ± 1.21	9.15 ± 1.33	16.1	15.6					

EF: *Epinephelus fuscoguttatus*; EH: *Epinephelus hexagonatus*.

### 2.3. Attenuated Total Reflectance-Fourier Transform Infrared Analysis (ATR-FTIR)

**Figure 3:**
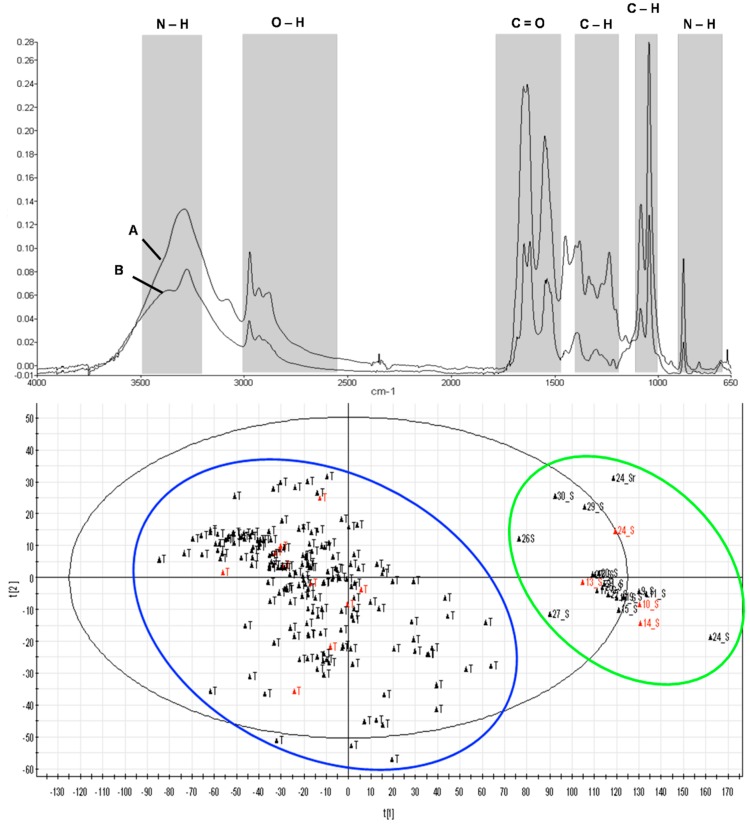
Two-dimensional plot using the first two principal components. Green cluster represents location from Sabah (A) and blue cluster represents location fromTerengganu (B). Red plot represents *E. hexagonatus* and black plot represents *E. fuscoguttatus*.

Quantitative FT-IR data for each sample are given in Figure 3. The PCA of these FT-IR data is displayed in a two-dimensional plot using the first two principal components (Figure 3). Three replicate samples of each individual were grouped in discrete clusters, indicating that PCA is able to discriminate individuals by sample and location. The FTIR spectrum (Figure 3) resulted in six functional groups consisting of amines, carboxylic acids, alkenes, and alcohols (Table 3). The patterns of absorptions are similar for both samples; however, the FTIR spectrum shows different intensities at the 3500–3300 cm^−1^ (Amines), 1700–1500 cm^−1^ (Carboxylic acids), and 1430–1290 cm^−1^ (Alkenes) regions.

**Table 3:** Functional Group of FTIR Spectrum.

Functional Group		Spectrum Region (cm^−1^)
Amines	N–H Stretch	3500–3300
Carboxylic acids	O–H Stretch	3000–2500
Carboxylic acids	C=O Stretch	1700–1500
Alkenes	C–H Bend	1430–1290
Alcohols	C–O	1260–1000
Amines	N–H Bend	~800

The score scatter plot divided the samples into two groups by location along PC1 and PC2. The samples from Terengganu were clustered on the right side of the plot, and the samples from Sabah (Borneo) were clustered on the left, which indicates that the ATR-FTIR is able to separate samples based on location. The separation of individuals in the score scatter plot was in agreement with known locations of the fish and also supports the findings of a previous study [10]. However, the score scatter plot could not separate *E. fuscoguttatus* from *E. hexagonatus*, indicating that the samples should be in one group. The score scatter plot results supported the phylogenetic tree results from molecular analysis. The non-separation of individuals in the score scatter plot was in agreement with the known molecular analysis of the fish. These results confirm the same putative introgressed sequences indicated by the phylogenetic reconstructions.

## 3. Discussion

### 3.1. Molecular Analysis

The congruent results by the MP and NJ analyses further reinforce our preliminary investigation that *E. fuscoguttatus* and *E. hexagonatus* form a monophyletic clade and that the population is phylogenetically closer to each other. We found no significant differences among these types of calibration/estimated nodes. The molecular data demonstrate that there is little genetic distance between the *E. fuscoguttatus* and *E. hexagonatus* (e.g., Kimura 2-parameter distance is 0.008 and 0.029) indicating a very close relationship on the molecular level. Phylogenetic inference by neighbor-joining and maximum-parsimony analysis revealed that *E. fuscoguttatus* and *E. hexagonatus* representing a similar taxon analysed either in COI, COIII or in 16S. This study also supported our previous phylogenetic relationships of *E. fuscoguttatus* and *E. hexagonatus* using cytochrome b mtDNA markers [10].

This study has revealed a remarkable level of similarity between the *E. fuscoguttatus* and *E. hexagonatus*, using several molecular markers. Our molecular analysis, supported by high bootstrap values for the *E. hexagonatus* node, clearly demonstrated that the species *E. fuscoguttatus* is misidentified as *E. hexagonatus* for which sequence divergence is very low. It has been demonstrated in other groups of fishes that some morphological characters are clearly adaptative to nature and that this may be affected by recurrent evolution in different lineages and caused confusion in identifying species that resulting in misidentification [12,13,14].

Moreover, several studies based on molecular data have indicated that phylogenetic relationships in fish are not always congruent with the phylogeny based on morphological data [15]. Molecular data examined suggest that the previous practice of identifying grouper species by morphological data may not be effective in species recognition and in discerning the true relationships within Epinephelinae spp. These 125 samples with six unique haplotypes of 16S, COI and COIII are identifiable using PCR techniques on a segment of mitochondrial 16S. The ability to distinguish different species based on muscle tissues when the external characteristics have been removed is of great commercial importance. The mitochondrial 16S, COI and COIII are commonly targeted for phylogenetic analysis, and several unique nucleotide positions that are useful to identify the respective species have been described.

### 3.2. Morphological Analysis

The biometric analysis, including the meristic and morphometric characteristics, has been adopted by many studies to identify and relate different fish species and populations [16]. This trend in biometric analysis reflects its validity in stock identification in different fisheries throughout the world. The present findings confirmed the validity of this biometric approach. Moreover, the type of allometry was used to study intra- and inter-specific variations in some fish species [17]. The present work confirms this statement and emphasises the taxonomic significance of allometric criterion in revealing intra- and inter-specific variations of morphometric characters of *E. fuscoguttatus* and *E. hexagonatus.* In the present study, the total ray counts and length measurements were reliable for the differentiation between *E. fuscoguttatus* and *E. hexagonatus*. All patterns of morphometric variations referred to *E. fuscoguttatus* as not clearly separated from *E. hexagonatus* because they shared the same number of rays and length. The dorsal, pectoral, pelvic and anal fin rays and spines were found to be constant on the generic level.

### 3.3. Attenuated Total Reflectance-Fourier Transform Infrared Analysis (ATR-FTIR)

The IR spectrum is measured by calculating the intensity of the IR radiation before and after it passes through a sample and the spectrum is traditionally plotted with the Y-axis units as absorbance or transmittance and the X-axis units as wave number units. For quantitative purposes it is necessary to plot the spectrum in absorbance units [18]. The intensity and, more accurately, the areas of the absorption peaks in the FTIR spectrum, were directly related to the concentration of the molecules. The spectrum is quite complex and contains several peaks arising from the contribution of different functional groups belonging to proteins, lipids and carbohydrates. Analysis was performed using a scanning process conducted on wavenumbers 4000–400 cm^−1^ with a resolution of 4 cm^−1^. The scanning results were percentage absorbance on the specific wavenumber for every functional group in each sample [19].

Each functional group has a specific marker group. Carbonyl groups as fatty acid markers were detected on wavenumber 1746 cm^−1^. The fatty acid absorbencies in every sample were used to compare relative protein levels in samples. The functional group was determined by comparing the wave numbers of functional groups. Various types of functional groups exist in each sample. All of the functional groups were identified by FTIR because of the specific mechanism that FTIR offers. It has been previously demonstrated that tissue proteins, carbohydrates and lipids play a role in the sensitivity of environmental effects. The infrared of the protein is characterised by a set of absorption regions: the amide region and the C–H region. The peaks arise principally from the C=O stretching vibration of the peptide group which is primarily N–H bending with a contribution from C–N stretching vibrations. This C=O peak is sensitive to the environment of the peptide linkage and also depends on the proteins overall secondary structure. The ratio of the peak intensities observed (1541 and 3297 cm^−1^) due to N–H bending and O–H stretching, respectively, could be used as indicators of the relative protein concentration and elucidate environmental effects on biological tissues [20].

The overall spectral profile is similar except for the variation in the intensities of the peak. The lower peak intensities represent samples from Terengganu and the higher peak intensities represent samples from Sabah. The peak at 3297 cm^−1^ is assigned to O–H stretching. The peaks observed at 2923 and 2853 cm^−1^ are due to the asymmetric and symmetric stretching of the membrane lipids, respectively. The other peak observed at 1653 cm^−1^ is assigned to the peak of C=O respectively. FT-IR analyses are often chosen by various studies because they are relatively fast, simple and require little or no sample preparation for spectral acquisition. Furthermore, the machine is non-destructive towards the sample cells/tissues because the samples remain intact during analysis. According to Chen *et al.* [21], FT-IR analysis is a universal method where the instruments and software are readily available and can be used for routine analysis as well as quantitative and qualitative analyses. The spectra provide information on the cell/tissue composition and the number of functional groups present.

Moreover, the FT-IR allows a multiple sample environment where samples can be in the form of liquid, gas, powder, solid, or film. Additionally, it is relatively less expensive for animals, plants and bacteria identification compared to several commonly used methods [22]. Multivariate statistical analysis of FT-IR spectra (chemometrics) can be divided into two types: supervised methods and unsupervised methods. The objective of unsupervised methods is to extrapolate the spectral data without prior knowledge of *E. fuscoguttatus* and *E. hexagonatus*. Principal component analysis (PCA) is an example of an unsupervised method.

PCA is used to reduce the multidimensionality of the data set into its most dominant components or scores while maintaining the relevant variation between the data points. PCA identifies the natural clusters in the data set with the first principal component (PC) expressing the largest amount of variation, followed by the second PC that conveys the second most important factor of the remaining analysis [23] and so forth. Score plots can be used to interpret the similarities and differences between *E. fuscoguttatus* and *E. hexagonatus*. The closer the samples are within a score plot, the more similar they are with respect to the principal component score evaluated. Studies using FT-IR have been utilised in plants [24,25,26], but few studies have been conducted with animals [27,28]. To date, no studies have been conducted on marine fish. This is the first study on marine fish using FT-IR to differentiate the origin and location of marine fish populations.

Misidentification due to homogenous nature leading to unrelated individuals being assigned to a full-sib family would not increase the inbreeding in the progeny. *E. hexagonatus*, *E. spilotoceps*, *E. macrospilos*, *E. howlandi*, *E. faveatus*, and *E. melanostigma* have always been frequently confused in the literature. They were often misidentified as other species since we know that grouper are highly stressed fish and when they become stressed, they tend to change their characteristics and camouflage to the surrounding environment [29].

If breeding with different species, especially with one that was not used as a food source, the outcome will be unfortunate for the farmers. Cross-breeding with wrong species will produce all sorts of wrong traits including growth retardation, slow feeding rate, low disease defenses and higher cannibalism amongst each other. This will result in death and high loss to the farmers. Criteria for choosing individuals that will be founders should be essentially the same as those used when the selection response is optimized under restricted co-ancestry when pedigree information is available. It is necessary to avoid mating between close relatives for managing existing quantitative genetic variation at the start of the programme [30]. Once it is clear that two species are conspecific, the cost for farm management and the rate of breeding between different species that will produce low value fish, low eggs production and low survival rate of the fry, is reduced [29]. Here, *E. fuscoguttatus* was misidentified as *E. hexagonatus* due to homogenous nature.

## 4. Experimental Section

### 4.1. Collection and Preservation

A piece of caudal fin area (fin clips at the edge of the fin) (20–50 mg) was collected from 125 individuals of grouper representing two taxa *i.e.*, *E. fuscoguttatus* and *E. hexagonatus*. The specimens were collected from five different locations of the Fisheries Research Institute, in Kampung Raja: 5.812213° N 102.589055° E, Kampung Seberang Timur: 5.991414° N 104.561976° E, Kampong Sentosa of Terengganu and 6.293416° N 105.61385° E Tanjung Badak: 6.201995° N 116.195526° E and Tuaran: 6.950465° N 121.144854° E of Sabah (Borneo) Malaysia were used for the molecular analysis. The samples were obtained from hatcheries and open sea cages. The specimens were then preserved in 95% ethanol at ambient temperature while in the field and at −20 °C under laboratory conditions. The specimens were homogenised in chilled nuclei lysis solution Wizard DNA isolation Kit (Promega, Madison, WI, USA) and kept at −20 °C. Samples for morphology and meristics were also collected at the same five locations of the same species.

### 4.2. DNA Extraction

DNA extraction was conducted using the Wizard DNA isolation Kit (Promega, USA) adapted for fin clip genomic DNA extraction.

### 4.3. Amplifying and Sequencing

Polymerase Chain Reaction (PCR) was used to amplify an approximately 450-bp fragment of the cyt b gene and an approximately 650-bp fragment of the 16S gene. Amplification reactions of 100 μL were prepared with 10–100 ng of DNA, 1.5 mM MgCl_2_, 2.5 U of *Taq* DNA polymerase, 200 mM dNTPs, and 0.1 mM of each primer.

The following step procedure was used to amplify the 16S gene following a 5 min denaturation at 94 °C: 94 °C for 1 min 30 s, 54.8 °C for 2 min and 72 °C for 1 min 30 s. A total of 30 cycles were performed using an Eppendorf MasterCycler (Hamburg, Germany). The following primer set was used to amplify the 16S gene: S16M: 5′-CGCCTGTTTATCAAAAACAT-3′ and S16KM: 5′-CCGGTCTGAACTCAGATCACGT-3′. Two portions of the mitochondrial genome were also amplified by polymerase chain reaction (PCR): an approximately 800 base pair (bp) region within the COI gene and an approximately 1500 bp region within the COIII gene. Primer sequences for COI were Co1FR 5′-CGCCTGTTTATCAAAAACAT-3′ and Co1RV 5′-GATATAAGAAGTCTAGCCTG-3′. Primer sequences for COIII were Co3FR 5′-GGAGGATTTGGAAATTGATTAGTTC-3′ and Co3RV 5′-GGGATAGCAATATTATGT-3′. These primers were designed based on sequenced samples using Primer-BLAST, NCBI (Bethesda, MD, USA) Primer3 [31]. PCR amplification was performed in 50 µL reactions containing 5 µL of 10× PCR buffer (Promega), 2.5 mM MgCl2, 0.2 µM dNTPs, 2–6 µL of template DNA, 0.5 µM of each primer, and 1.25 units of Taq polymerase. Reactions were performed with an initial denaturation step at 94 °C for 3 min, then 30–40 cycles of 94 °C for 30 s, 53.2 °C for 30 s, 50 °C for 90 s, 72 °C for 120 s, and a final extension step at 72 °C for 10 min. Negative controls were performed for all amplification reactions. In addition, PCR and sequencing were repeated to confirm putative introgressed sequences and to exclude the possibility that they were the result of PCR contamination. No identification problems were found.

PCR amplification products were separated on a 1% agarose gel stained with ethidium bromide (EtBr). Products of interest were identified using a *Hin*dIII DNA ladder as a reference marker (Promega, USA). PCR fragments were purified from the gel using a Wizard-Prep PCR Purification Kit (Promega). Automated fluorescent dideoxy sequencing of both strands was carried out using an ABI Prism 3100 DNA sequencer (Applied Biosystems, Foster City, CA, USA) at the available sequencing centre and we provide our own 16S, COXI and COXIII primers for the sequencing services. Sequences from both strands of all the individuals from each species were assembled into a single consensus sequence using the assembly editor option in the Biology Workbench (San Diego, CA, USA) (ver. 3.2) [32]. The consensus sequences were aligned using the alignment program Clustal W with the default settings [33].

### 4.4. Data Analysis

The DNA sequence data for all samples were analysed for ambiguities and the nucleotide sequences obtained were aligned by ClustalX2 [34] with default settings. Only unique haplotype was chosen for the phylogenetic analysis. Because variation within the nuclear loci was low, relationships between sequences for each locus were determined, and the data set was analysed with maximum parsimony (MP), molecular evolution (ME), and neighbour-joining (NJ) methods using the program MEGA 4.0 [35]. Sites with missing data were removed, and the mitochondrial region sequences were used to test models of evolution. The Kimura 2-parameter model of evolution was employed to test the MP, ME and NJ trees. Kimura 2-parameter model corrects for multiple hits, taking into account transitional and transversional substitution rates, the four nucleotide frequencies are the same and that rates of substitution do not vary among sites [36].

The haplotype were analysed with MP, ME and NJ as implemented DNAsp Version 5 [37]. Support for nodes was estimated using the bootstrap technique with 1000 replicates using AY950700 and DQ067314 from *Epinephelus bruneus* as an outgroup. All new sequences were deposited in GenBank, as detailed in Supplementary Table S1. The haplotype and the nucleotide diversity were calculated in order to examine the levels of gene variability within samples and gene genealogy when dealing with large data sets of closely related species; for this purpose Arlequin Ver 3.5 [38] and TCS v1.21 [39] were used. The neutrality value in the samples was assessed by calculating Tajima’s D value using 1000 permutations in the Arlequin Ver 3.5.

### 4.5. Morphological Analysis

Wild species of *E. fuscoguttatus* and *E. hexagonatus* specimens were caught by fishing nets in ponds in the coastal and open South China Sea. Ten specimens (four males and six females) were collected at the five locations, two specimens from each location. The total length of the analysed specimens ranged from 58.0 to 61.0 cm and the weights ranged from 6.0 to 8.5 kg. All morphometric and meristic characteristics were examined according to Heemstra and Randall [40] by colour patterns and the scale of rays, fins, and spines as diagnostic characters for identification. The specimens were measured with a digital slide caliper (24 inches measuring range) and weighed with a high capacity electric balance (12,000 g × 0.1 g).

### 4.6. Attenuated Total Reflectance-Fourier Transform Infrared Analysis (ATR-FTIR)

Fin tissues of samples were subjected to FTIR analysis. The freeze-dried fin samples were placed directly on the diamond window. All samples were oriented in the north-south configuration and aligned with the probing beam to minimise unwanted spectral differences due to sample placement. The micrometer accessory had a straight-edged metal tip attachment and each sample was collected while applying approximately 800 psi (55 bar) of pressure (PerkinElmer, Boston, MA, USA). The analysis was conducted using an FT-IR spectrometer (PerkinElmer, USA) equipped with a DTGS detector. MIR spectra were recorded from an accumulation of 4 scans in the 4000–400 cm^−1^ range.

## 5. Conclusions

This study has provided important molecular, morphological and spectroscopy information that can be used to identify the *E. fuscoguttatus* species more precisely. Additionally, the study has successfully proven the utility of molecular, morphological and spectroscopy techniques in identifying *E. fuscoguttatus.* This study found that *E. fuscoguttatus* has been misidentified to *E. hexagonatus*. The variation in color pattern and morphology of *E. hexagonatus* that have been observed are likely attributed to differences in environment or nature of the fish’s habitat such as the coral reef; it is well known that the grouper family can adapt and camouflage in order to protect themselves [9]. It could be that these two taxa are in fact undergoing speciation, however the process is not complete and, therefore, they are currently occupying separate environmental niches, leading to difference in morphology, but are capable of interbreeding, and that *E. fuscoguttatus* was misidentified as *E. hexagonatus*. The former name takes precedence over *E. hexagonatus* as it was described first. They may form two separate species in the future if the populations become reproductively isolated. The potential of molecular genetics as one of the techniques in identification of grouper species should be considered for establishing proper management and breeding strategies for the valuable *Epinephelus fuscoguttatus* species in Malaysia.

## Supplementary Materials

Supplementary materials can be found at http://www.mdpi.com/1422-0067/16/07/14884/s1.
