# Supplementary material for: Homogeneous Nature of Malaysian Marine Fish Epinephelus fuscoguttatus (Perciformes; Serranidae): Evidence Based on Molecular Markers, Morphology and Fourier Transform Infrared Analysis
Source: Int J Mol Sci. 2015 Jul 2;16(7):14884–900. doi: 10.3390/ijms160714884 (PMC4519877; doi:10.3390/ijms160714884)
Supplement: Supplementary file 1 [file ijms-16-14884-s001.pdf]

Supplementary Information

Table S1. List of taxa sequenced of Cytochrome b and 16S and their GenBank accession numbers.

	Species	Label	GenBank ID
**Cytochrome b**	*Epinephelus hexagonatus*	*3C11*	GU591708
	Epinephelus hexagonatus	*3C27*	GU591711
	Epinephelus hexagonatus	*4C7*	GU591702
	Epinephelus hexagonatus	*4C10*	GU591703
	Epinephelus hexagonatus	*4C13*	GU591704
	Epinephelus hexagonatus	*4C17*	GU591705
	Epinephelus hexagonatus	*5C18*	GU591718
	Epinephelus hexagonatus	*6C6*	GU591720
**16S**	*Epinephelus hexagonatus*	*3s11*	HQ840441
	Epinephelus hexagonatus	*3s20*	HQ840443
	Epinephelus hexagonatus	*4s1*	HQ840444
	Epinephelus hexagonatus	*4s7*	HQ840445
	Epinephelus hexagonatus	*4s17*	HQ840446
	Epinephelus hexagonatus	*4s18*	HQ840447
	Epinephelus hexagonatus	*5s18*	HQ840449
	Epinephelus hexagonatus	*6s9*	HQ840450
